# Open-bore MRI Scanner Assessment of Epicardial Adipose Tissue after Bariatric Surgery: A Pilot Study

**DOI:** 10.2174/0118715303310680240607114244

**Published:** 2024-08-21

**Authors:** Carmela Asteria, Francesco Secchi, Lelio Morricone, Alexis Elias Malavazos, Simona, Francesconi, Valentina Milani, Alessandro Giovanelli

**Affiliations:** 1 National Institute of Obesity Cure (INCO)-Bariatric Unit, IRCCS, Policlinico San Donato, Piazza Edmondo Malan, 2, 20097, San Donato Milanese, Milan, Italy;; 2 Department of Biomedical Sciences for Health, Università degli Studi di Milano, Via Mangiagalli 31, 20133, Milano, Italy;; 3 Department of Radiology, IRCCS Policlinico San Donato, piazza Edmondo Malan, 2, 20097, San Donato Milanese, Italy;; 4 Head of Cardiovascular Imaging, IRCCS Multimedica, via Milanese, 300, Sesto San Giovanni, 20099, Milan, Italy;; 5 Metabolic Diseases Service, Palazzo della Salute, Gruppo San Donato (GSD), via Teodorico, 25, 20149, Milan, Italy;; 6 Endocrinology Unit, Clinical Nutrition and Cardiovascular Prevention Service, IRCCS, Policlinico San Donato, piazza Edmondo Malan, 2, 20097, San Donato Milanese, Milan, Italy;; 7 Laboratory of Biostatistics and Data Management, Scientific Directorate, IRCCS, Policlinico San Donato, San Donato Milanese, 20097 Milan, Italy

**Keywords:** Epicardial adipose tissue (EAT), visceral adipose tissue (VAT), laparoscopic sleeve gastrectomy (LSG), laparoscopic roux-en-Y gastric bypass (RYGBP), metabolic syndrome (MS), open-bore magnetic resonance imaging (MRI)

## Abstract

**Background:**

The recognition of epicardial adipose tissue (EAT) as a cardiac risk factor has increased the interest in strategies that target cardiac adipose tissue.

**Aim:**

The effect of bariatric and metabolic surgery (BMS)-induced weight loss on EAT volume was evaluated in this study.

**Methods:**

Fifteen bariatric patients, with (MS) or without (wMS) Metabolic Syndrome, underwent magnetic resonance imaging (MRI) using an open-bore scanner to assess EAT volume, visceral adipose tissue (VAT) thickness, and other cardiac morpho-functional parameters at baseline and 12 months after BMS. Nine patients underwent laparoscopic sleeve gastrectomy (LSG), and 6 patients underwent Roux-en-Y Gastric Bypass (RYGBP).

**Results:**

EAT volume significantly decreased in all the patients 12 months post-BMS from 91.6 cm^3^ to 67.1 cm^3^*; p* = 0.0002 in diastole and from 89.4 cm^3^ to 68.2 cm^3^*; p* = 0.0002 in systole. No significant difference was found between the LSG and RYGBP group. Moreover, EAT volume was significantly reduced among wMS compared with MS. In particular, EAT volume in diastole was significantly reduced from 80.9 cm^3^ to 54.4 cm^3^; *p* = 0.0156 in wMS and from 98.3 cm^3^ to 79.5 cm^3^; *p* = 0.031 in MS. The reduction was also confirmed in systole from 81.2 cm^3^ to 54.1 cm^3^; *p* = 0.0156 in wMS and from 105.7 cm^3^ to 75.1 cm^3^; *p* = 0.031 in MS. Finally, a positive correlation was found between EAT loss, BMI (*r* = 0.52; *p* = 0.0443) and VAT (*r* = 0.66; *p* = 0.008) reduction after BMS.

**Conclusion:**

These findings suggest that EAT reduction may be a fundamental element for improving the cardio-metabolic prognosis of bariatric patients. Moreover, this is the first study performed with an open-bore MRI scanner to measure EAT volume.

## 1 INTRODUCTION

Epicardial adipose tissue (EAT) is the true visceral adipose tissue (VAT) of the heart located between the myocardium and visceral pericardium, surrounding the coronary vessels [1-4]. It is a quantifiable, modifiable, and multifaceted tissue that has both local and systemic effects. Its thermogenic functions depend on the nature of “beige” adipose tissue [5, 6]. Several studies have increasingly highlighted how EAT represents much more than a simple storage site for adipose tissue, showing numerous endocrine, paracrine, and vasocrine interplays with the neighbouring structures [7-11]. Even though EAT physiologically produces anti-inflammatory cytokines such as adiponectin and provides antiatherogenic and cardioprotective effects, in pathological conditions, it may produce proinflammatory cytokines and promote the development of coronary artery disease (CAD) [12, 13], as in the case of obesity and its metabolic consequences [10, 11]. In these particular conditions, there is an increase in EAT volume due to inflammation [14, 15], and such changes may be regarded as an early biomarker of cardiometabolic risk [15, 16]. Notably, in the obese population EAT may act as a transducer of systemic inflammation and metabolic dysregulation from the whole body to the heart [17]. Moreover, EAT strongly and independently reflects the intra-abdominal visceral fat [18] and intra-myocardial lipid content [19]. Epicardial fat thickness is also significantly related to metabolic syndrome [20, 21] and other traditional cardiometabolic risk factors [22-25].

The recognition of EAT as a cardiac risk factor has increased the interest in strategies that target cardiac adipose tissue [26-28]. There are few studies that evaluate the effect of weight loss on the amount of EAT in subjects with obesity [29, 30]. Other studies indicate that EAT volume can be modified by pharmaceutical interventions, including both GLP-1 analogues [31-33], sodium-glucose co-transporter inhibitors (SGLT2i) [34, 35], and statins [35-37]. However, the treatment with the greatest impact on severe obesity is bariatric and metabolic surgery (BMS), which guarantees sustained weight loss and reduces cardiovascular mortality and cardiovascular events by 50% [38]. Although the weight loss obtained following BMS is able to reduce visceral fat and other depots of adipose tissue, the effects of BMS on EAT have not been adequately characterized. It has been hypothesized that ectopic depots of adipose tissue in the heart can be mobilized in patients undergoing BMS, but studies have often shown conflicting results [39-44].

In line with Rabkin and Campbell [45], a recent systematic review found in eight of nine studies that BMS was significantly associated with a reduction of EAT amount ranging from 5.3% to 31.3% [46-53].

One study did not find a significant change in EAT volume at 3 months after BMS [46].

Weight loss following BMS and related reduction in visceral fat may have a favorable effect on cardiac structure and function even in long-term evaluation. After a long follow-up period, EAT thickness was reduced by 14% [54]. However, only four of nine studies evaluated the EAT volume. The remaining studies evaluated EAT thickness by echocardiography.

The EAT volume can be assessed *via* non-invasive imaging studies such as computed tomography (CT) and magnetic resonance imaging (MRI) [55-61].

Both techniques allow the estimate of the EAT volume on routine scans without the need for contrast agent injection. In particular, MRI, which does not expose the patient to ionizing radiation, allows the assessment of EAT on cine bright-blood, steady-state free precession images, which are a mainstay in every routine cardiac MRI examination.

However, MRI assessment of EAT in obese patients is challenging, as the bore of MRI scanners may not be sufficiently wide to accommodate large sizes [62]. A potential solution for this issue is the use of open-bore MRI scanners, which have been shown to provide at least a subjective image quality comparable to that provided by closed-bore scanners [63]. Nevertheless, data concerning the accuracy and precision of quantifying EAT using images acquired from open-bore MRI scanners are still scarce. For this purpose, our group has recently published a paper that suggests a good intra- and inter-reader reproducibility of EAT volumes, measured on scans performed by an open-bore MRI scanner, in a population of patients with severe obesity suitable for BMS [64].

The aim of the present study was to measure the EAT volume and other cardiac morpho-functional parameters in subjects suffering from severe/extreme obesity, with or without Metabolic Syndrome (MS), referred to our Hospital for BMS, using an MRI scanner with an open conformation capable of supporting up to 220 kg of weight. MRI was performed one month before surgery (T0) and one year after BMS (T1).

The variation between different types of BMS, such as laparoscopic gastric by-pass according to Roux-en-Y (RYGBP) or laparoscopic sleeve gastrectomy (LSG), was considered in order to identify whether, for the same weight loss, there were different effects on the EAT volume. The presence of MS was considered a possible negative factor in the reduction of EAT volume.

This is a multicentre prospective observational study and involves The Policlinico San Donato Research Hospital (IRCCS), Sano Donato Milanese, Milan, Italy, as the promoting Centre in collaboration with the Centro Diagnostico Italiano, Milan, Italy.

## 2 MATERIALS AND METHODS

The ethical approval was obtained for both Centres (Ethics Committee of San Raffaele Research Hospital approved the study on May 11^th^, 2017; protocol code and number: *EAT-BS; 151/INT/2026*). All subjects signed a dedicated informed consent.

### 2.1 Study Population

Out of 17 initially enrolled patients, 15 subjects with severe/extreme obesity, 2 males and 13 females, aged between 18 and 65 years, were referred for BMS at the Bariatric and Metabolic Surgery Unit of the INCO (National Institute for Obesity Treatment), Policlinico San Donato, completed the study and 2 were excluded due to the excessive size for the MRI assessment. All patients underwent BMS: LSG or RYGBP.

They were preliminarily evaluated by a multidisciplinary team to establish compliance with the criteria suggested by the guidelines for bariatric surgery (SICOB) and were recruited for standard indication: the body mass index (BMI) was ≥ 40 kg/m^2^ or ≥ 35 kg/m^2^ in the presence of comorbidities (cardiovascular, respiratory or metabolic pathologies, severe articular pathology or psychological comorbidities).

Exclusion criteria were the presence of overt cardiac pathologies such as ischemia and valvular disease or contraindications to BMS: patients <18 years or >65 years of age or with cardiac, renal, or hepatic failure, poorly controlled diabetes mellitus, hypertension, neoplastic diseases, pregnancy and major psychiatric disorders or unstable eating disorders.

Moreover, patients with the main contraindications to MRI imaging, namely unsafe or conditional devices, intracranial ferromagnetic clips, intraocular metallic chippings, severe claustrophobia, impossibility of maintaining a supine position or avoiding involuntary movements, and heavily artifacted MRI images were excluded from the analysis. The sager guidelines were followed.

### 2.2 Anthropometric Parameters

At baseline and 12 months after BMS, anthropometric parameters were obtained by the treating physician, including age, sex, weight, height, BMI, waist circumference (WC), and waist-to-height ratio (WHtR). This index is defined as WC, usually measured midway between the lower rib and the iliac crest, divided by height, both measured in the same units (cm). Weight and BMI were collected using an electronic body composition analyzer (Seca mBCA 515, Intermed Srl, Milan, Italy) with weight capacity of up to 300 Kg.

### 2.3 Blood Tests

All patients underwent blood testing one month before surgery (T0) and 12 months after surgery (T1) for the diagnosis of Metabolic Syndrome (MS) based on the National Cholesterol Education Program/Adult Treatment Panel III criteria (NCEP/ATPIII, 2002) [65]. Total cholesterol (TC), high-density lipoprotein cholesterol (HDL-C), triglycerides (TGs), HbA1c, and fasting blood glucose (FBG) levels were measured.

Moreover, the lipid accumulation product (LAP) was obtained using the formula proposed by Kahn: for men = (WC [cm] − 65) × (TG [mmol/L]); for women = (WC [cm] − 58) × (TG [mmol/L]) [66].

Blood samples were drawn after overnight fasting, and all the examinations were carried out by the Policlinico San Donato clinical biochemistry laboratory according to standardised protocols the data obtained were the results of the latest blood biochemical examination in the last three months. The cut-off points used for each parameter were the following: TC ≥200 mg/dL, TGs ≥150 mg/dL, LDL-C ≥120 mg/dL, and FBG ≥110 mg/ml.

### 2.4 Cardiological Examinations

All patients had a detailed physical examination and blood pressure measurement, and all were screened for cardiovascular risk factors. A normal 12-lead electrocardiogram and normal global and regional resting cardiac function were assessed by transthoracic echocardiography.

### 2.5 Image Acquisition

All enrolled subjects underwent the following assessments at T0 and T1 by MRI cardiac scan. MRI examinations, without contrast medium, were performed using a 1.0-T open-bore scanner (Panorama, Philips Medical Systems, Best, The Netherlands), equipped with 26 mT/m gradient power, using either an 8-channel surface phased array coil (SENSE Body-L, Philips Medical Systems, Best, The Netherlands) or a 3-channel surface phased array coil (SENSE Body-XL, Philips Medical Systems, Best, The Netherlands), depending on the size of the patient. For each patient, an electrocardiographic (ECG) triggered cine steady-state free precession sequence was acquired in a short axis covering the heart from the apex to the basal portion, with the following parameters: slice thickness 10 mm, field of view 320 × 100 mm^2^, flip angle 70°, time 132 of repetition 4.7 ms and time of echo 2.1 ms.

Moreover, a retrospectively ECG-triggered breath-hold two-dimensional phase-contrast gradient recalled echo sequences with a through-plane velocity encoding gradient ranging from 150 to 350 cm/s were performed on a transverse plane above the aortic bulb. Sequence parameters were as follows: repetition time 49.75 ms, echo time 3.1 ms, flip angle 30°. Retrospective ECG-gating with 30 phases per cycle (with repetition time dependent on the R-R interval) was set.

### 2.6 Image Analysis

For each patient, the pericardium was segmented on short-axis cine images by a reader with a 4-year experience in cardiovascular MRI, who manually traced the contour of the pericardium on every slice, using a freeware software, ITK-SNAP Version 3.8.0 (www.itksnap.org) [67], as depicted in Fig. (**1**), both on systolic and diastolic frames which were previously chosen by the reader.

Afterward, the reader traced the contour of the epicardium to exclude tissues different from EAT, which were included in the previous segmentation, as depicted in Fig. (**1**). Along the longitudinal cardiac axis, the segmentation included slices from the cardiac base to its apex. The EAT volume was obtained by multiplying the areas by the slice thickness slice by slice, then summing all the values to obtain a measurement expressed as cm^3^.

Moreover, the thickness of the visceral adipose tissue (VAT) was also measured near the superior splenic pole in the bright blood axial scout sequences.

### 2.7 Volume Analysis and Flow Analysis

A radiologist with 15 years of experience performed the segmentation of cardiac and flow images using MEDIS QMass 7.6 and QFlow 5.6 (Medis Medical Imaging Systems, Leiden, The Netherlands).

For the segmentation of cardiac images, the reader manually traced the epicardial contour of both LV and RV in the end-diastolic and end-systolic phases. Then, the software applied the blood threshold technique (Mass-K mode) and calculated the end-diastolic volume (EDV), end-systolic volume (ESV), stroke volume (SV), ejection fraction (EF), and cardiac mass values in a totally automated way. A 50% threshold was fixed using Mass-K mode.

For the segmentation of flow images of the ascending aorta, the reader positioned a region of interest on the vessel boundary of a selected slice. Subsequently, the reader propagated the segmentation through the remaining slices, and the software proposed an automated adjustment, in addition, the reader was allowed to manually correct the adjusted contours. For each session, maximum and minimal area and forward and backward flow measurements were obtained. The aortic Area Strain (AAS) was also calculated as (Amax - Amin)/Amin [68].

### 2.8 Statistical Analysis

Categorical data were reported as number and frequency. Continuous data were reported as mean ± standard deviation or as median and interquartile range according to the distribution of the variable. Normal data distribution was assessed through the Shapiro-Wilk test, and differences between patients with or without MS and type of BMS were computed through the Mann-Whitney test.

The median of EAT (primary outcome) and anthropometric, biochemical characteristics, volume, and cardiac morpho-functional parameters (secondary outcomes) before and after BMS were compared by nonparametric Wilcoxon signed-rank test. The Fisher’s exact test or Chi-square test was used to compare categorical data.

Correlations between continuous variables were evaluated according to Spearman Rho.

Given the small size of this pilot study testing, the interaction between the covariates could not be taken into account.

The statistical analysis was performed using SAS 9.4 (SAS Institute, Cary, NC). A 2-tailed *p* ≤ 0.05 was considered statistically significant.

## 3 RESULTS

### 3.1 Demographic, Anthropometric and Biochemical Characteristics of the Study Population

A total of 15 subjects with severe/extreme obesity completed the study: 2 men (13.3%) and 13 women (86.7%), median age 47 years, and median BMI 41.2 kg/m^2^. All patients underwent BMS: 9/15 SG (60%) and 6/15 RYGBP (40%). Their anthropometric and biochemical characteristics are shown in Table **1**. On the basis of blood testing and hypertension, 7/15 (46.7%) patients (5 females and 2 males) showed the presence of MS, according to the NCPE-ATPIII [65].

### 3.2 Changes in the Metabolic Profile (Anthropometric and Biochemical Parameters) after BMS

BMS reduced weight significantly from 107 kg to 73 kg (*p* <0.0001) and BMI from 41.2 kg/m_2_ to 24.1 kg/m_2_ (*p* <0.0001). There was also a significant decrease in waist circumference (from 118 cm to 84.3 mg/dl; *p* <0.0001). Both WHtR and LAP were reduced (from 0.74 cm to 0.54; *p* <0.0001 and from 73.95 cm x mmol/L to 22.79 cm x mmol/L; *p* <0.0001). As expected, after 12 months, BMS induced a significant improvement of FBG (from 101 mg/dl to 88 mg/dl; *p* = 0.0037) and HDL-C (from 49 mg/dl to 65 mg/dl; *p* = 0.0073). There was also a significant decrease in TGs (from 127 mg/dl to 76 mg/dl; *p* = 0.0139). Both WHtR and LAP were reduced (from 0.74 cm to 0.54; *p* <0.0001 and from 73.95 cm x mmol/L to 22.79 cm x mmol/L; *p* <0.0001) (Table **2**).

### 3.3 Changes in EAT Volume and VAT Thickness after BMS

EAT volume significantly decreases in all the patients after 12 months post-BMS (from 91.6 cm^3^ to 67.1 cm^3^; *p* = 0.0002) in the diastolic phase and (from 89.4 cm^3^ to 68.2 cm^3^; *p* = 0.0002) in the systolic phase. Moreover, EAT volume in the diastolic phase was reduced more in patients undergoing RYGBP than in those undergoing SG (from 94.4 cm^3^ to 57.9 cm^3^; *p* = 0.0313 in RYGBP and from 91.3 cm^3^ to 68.6 cm^3^; *p* = 0.0156 in SG). Similarly, the thickness of VAT significantly decreases in all the patients 12 months after the surgical procedure (from 6.45 mm to 3.2 mm; *p* = 0.00107) (Table **3**).

As shown in Fig. (**2**), a positive correlation between EAT volume in the diastolic phase and the reduction of BMI (r = 0.52; *p* = 0.0443) and VAT (r = 0.66; *p* = 0.0088) after the surgical procedure was found.

### 3.4 Changes in Cardiac Morpho-functional Parameters after BMS

A significant increase of LV EDVi (End-Diastolic Volume index) was observed in all the patients, from 54 ml/m^2^ to 60.2 ml/m^2^; *p* = 0.0009. An increase in RV volumes and mass was observed. In particular, RV EDVi increased from 49.4 ml/m^2^ to 67.5 ml/m^2^; *p* = 0.0015, RV ESVi from 17 ml/m^2^ to 24.8 ml/m^2^; *p* = 0.0006 and mass from 29 g/m2 to 38.2 g/m^2^; *p* = 0.0001. For both LV and RV, EF and SV remained unchanged. No significant increase in the maximum and minimum aortic area and AAS was observed (Table **4**).

### 3.5 Comparison between Changes in the Metabolic Profile (Anthropometric and Biochemical Parameters) after BMS in Patients with and without MS

BMI was reduced in patients with and without MS (respectively from 40.9 kg/m^2^ to 29.6 kg/m^2^; *p* = 0.0156 in MS and from 41.6 kg/m^2^ to 23.7 kg/m^2^; *p* = 0.0078 in wMS) (Table **5**). There was also a significant decrease of WC (from 121 cm to 79 cm; *p* = 0.0156 in MS and from 113.5 cm to 85.1 cm; *p* = 0.0078 in wMS), WHtR (from 0.74 cm to 0.49 cm; *p* = 0.0156 in MS and 0.71 cm to 0.54 cm; *p* = 0.0078 in wMS) and LAP (from 122.34 cm x mmol/L to 21.10 cm x mmol/L; *p* = 0.0156 in MS and from 49.30 cm x mmol/L to 23.41 cm x mmol/L; *p* = 0.0078 in wMS).

Patients wMS showed a significant decrease of glycemic levels (from 92.5 mg/dl to 86 mg/dl; *p* = 0.0469). BMS induced a significant increase of HDL-C (from 44 mg/dl to 61 mg/dl; *p* = 0.031) (Table **6**).

### 3.6 Comparison between Changes in EAT Volume and VAT Thickness after BMS in Patients with and without Metabolic Syndrome

As shown in Table **6**, EAT volume, both in diastole and in systole, significantly decreases in wMS compared with MS after 12 months post-BMS. In particular, EAT volume in the diastolic phase was significantly reduced from 80.9 cm^3^ to 54.4 cm^3^; *p* = 0.0156 in wMS and from 98.3 cm^3^ to 79.5 cm^3^; *p* = 0.031 in MS. The reduction was also confirmed in the systolic phase (from 81.2 cm^3^ to 54.1 cm^3^; *p* = 0.0156 in wMS and from 105.7 cm^3^ to 75.1 cm^3^; *p* = 0.031 in MS). Also, the thickness of VAT significantly decreased both in MS and wMS 12 months after the surgical procedure (from 7.0 to 4.0; *p* = 0.036 in MS and from 5.0 to 3.0; *p* = 0.021 in wMS) without a significant difference between the two groups.

### 3.7 Comparison between Changes in Cardiac Morpho-functional Parameters after BMS in Patients with and without Metabolic Syndrome

Taking into account the cardiac outcomes, an increase of LV EDVi was observed (from 41 ml/m^2^ to 60.2 ml/m^2^; *p* = 0.0156 in MS and from 54.1 ml/m^2^ to 63.5 ml/m^2^; *p* = 0.0547 in wMS), as well as an increase of RV volumes. In particular, RV EDVi increased from 47 ml/m^2^ to 62.2 ml/m^2^; *p* = 0.0156 in MS and from 54 ml/m^2^ to 70.3 ml/m2; *p* = 0.0781 in wMS, RV ESVi from 14 ml/m^2^ to 18.2 ml/m2; *p* = 0.0469 in MS from 18 ml/m^2^ to 27.6 ml/m^2^; *p* = 0.0156 in wMS. The mass increased from 31 g/m2 to 38.2 g/m^2^; *p* = 0.0156 in MS and from 27.8 g/m^2^ to 38.5 g/m^2^; *p* = 0.0156 in wMS (Table **7**).

## 4 DISCUSSION

EAT is located directly on the myocardium and around the coronary arteries and is believed to mediate inflammation, atherosclerosis, and myocardial dysfunction through paracrine signaling [7, 69]. The recognition of EAT as a novel cardiac risk factor has increased the interest in strategies that target cardiac adipose tissue. EAT can easily be assessed by volumetric analysis or by simple linear thickness measurements. A recent study by Aitken-Buck *et al.* [70] has suggested that the expansion of EAT is different from that of the visceral and subcutaneous adipose tissue: hyperplasia is the primary remodeling mechanism for EAT, whereas hypertrophy is the primary mechanism for visceral and subcutaneous adipose tissue. It is, therefore, expected that visceral, subcutaneous, and epicardial fat depots will respond differently to weight-loss interventions, and future investigations are necessary to evaluate to what extent the physiology and metabolic functions of EAT are affected by this [70].

Studies indicate that EAT volume can be reduced by BMS (22,30,31,45,46,47). These studies mostly consisted of SG (n = 1) or RYGBP (n = 3) or both (n = 5). Most studies (n = 5 of 9) evaluated the changes in cardiac adipose tissue 6 months after the surgery, but the duration from time of surgery to follow-up ranged from 3 months to 2 years. Eight of the nine studies investigating the effect of BMS on cardiac adipose tissue volume reported significant reductions in EAT volume ranging from 5.3% to 31.3% [46-53]. One study did not find a significant change in EAT volume at 3 months after BMS [71]. This result was confirmed 3 and 4 months after BMS by subsequent reports [52, 70]. The issue of whether different types of BMS produce different effects on EAT reduction despite similar reductions in weight has been raised by Kokkinos *et al*., who reported that although weight loss or reduction in WC was similar, there was a significant difference in terms of EAT reduction between Roux-en-Y and sleeve gastroplasty with a greater reduction in EAT with the Roux-en-Y procedure [51]. However, our study does not appear to support this contention. We found a significant reduction of EAT volume in both the diastolic and systolic phases 12 months after BMS, which was not related to the type of intervention. Another interesting finding was the significant positive correlation between EAT loss and BMI after the surgical procedure. In contrast, Gaborit *et al*. had most of their procedures using sleeve gastroplasty (19 of 23) and reported a significant and substantial reduction in EAT (-39 mL, *p* value < 0.001) [48], but they observed that “Unexpectedly, the percentage of EAT loss was not correlated to the percentage of BMI”. Also, in the paper of Wu *et al*., the percentage of EAT loss did not correlate with the percentage of BMI loss after BMS [46]. The apparent discrepancy may be due to the short period of observation which was of 3 or 4 months instead of 12 months, as in our study.

We also evaluated the correlation between EAT loss and the reduction of other clinical indicators of obesity, such as weight, WC, and WHtR [72]. This index is defined as WC, usually measured midway between the lower rib and the iliac crest, divided by height, both measured in the same units (cm). A 2010 systematic review concluded that “WHtR may be advantageous because it avoids the need for age-, sex- and ethnic-specific boundary values” [73]. It can be used as a predictor of obesity-related cardiovascular disease. A WHtR of over 0.5 is critical and signifies an increased risk. No correlation was found between WC and abdominal obesity, indirectly measured by the WhtR. However, a positive correlation was observed with VAT. According to the literature 30, this latter finding strongly suggests that EAT is a true visceral district, albeit with its peculiarities, and its reduction is a fundamental element for improving the cardio-metabolic prognosis of these patients.

As far as the influence of MS on EAT loss, we found that EAT volume 12 months after the surgical procedure was significantly reduced among wMS patients compared with the MS group. Moreover, EAT did not correlate significantly with any of the nonobesity components of the metabolic syndrome, specifically FBG, TC, TGs, and HDL-C. Therefore, the magnitude of the relationship of EAT to MS is considerably and significantly less than the relationship of EAT to BMI. This finding raises the possibility that the association of EAT with CAD cannot be readily explained by standard risk factors, and the unique features of this adipose tissue warrant further investigations.

In order to evaluate other markers of CAD and metabolic disorders [74] and their relationship with EAT, we measure the Lipid Accumulation Product (LAP). LAP is based on a combination of two variables: WC and fasting concentration of triglycerides (TGs). The simultaneous use of biochemical and anthropometric measurements to calculate LAP allows us to better describe both anatomical and biochemical changes related to lipid overaccumulation in humans.

However, no significant correlations were found after BMS between EAT volume and LAP in SG and RYGBP, nor were they found in MS and wMS patients. This finding could be due to the small number of patients in each subgroup.

This lack of correlation with MS could be explained by the fact that we measure EAT quantity and not its functional inflammatory status. Even though several studies provide evidence that EAT can be modified by different weight-loss strategies, as well as BMS, it is not fully understood whether reducing EAT volume is clinically relevant. A study by Oikonomou *et al*. [75] has suggested that the functional inflammatory status of EAT may be more clinically relevant than merely the EAT quantity. Future studies are warranted to determine whether modulation of EAT will translate into clinically relevant cardiovascular risk reductions and whether the volume or inflammation of EAT (or some other measures of EAT functional status) is the better cardiac risk measure.

As far as the cardiac morpho-functional variables are concerned, a significant increase of EDVI and ESVI for both LV and RV and ESVI and mass for RV was observed. Similar results were found in patients with MS. This increase could be due to the significant reduction of body weight after the procedure and seems to suggest a favorable remodeling of RV and LV after BMS, leading to an improvement of cardiac function. No significant changes were observed for LV and RV functional parameters or ascending aortic strain.

There are some limitations in this study, including, first of all, the small sample size and the disproportion between males and females. Another limitation regards the impossibility to account for one single analysis of the conditions to consider (type of surgery, metabolic syndrome, before and after bariatric surgery). Further evidence is necessary to better understand whether EAT may act as a transducer of systemic inflammation and metabolic dysregulation from the whole body to the heart.

Second, we did not evaluate the blood or tissue levels of proinflammatory adipokines or address the underlying mechanisms of change in EAT. Therefore, the lack of correlation with MS could be explained by the fact that we measure EAT quantity and not its functional inflammatory status.

Despite these limitations, in this study, EAT volume was quantified, as far as we know, for the first time by using an open-bore MRI scanner that was sufficiently wide to accommodate large sizes who otherwise could not receive adequate imaging examinations. The accuracy and precision of quantifying EAT by this approach were demonstrated by our group in a recent publication [51]. Our data are comparable to those obtained by close-bore MRI scanners in other studies. Another important strength is that our study focused on EAT and did not include pericardial fat, avoiding problems with data interpretation when combining two different fat depots in the evaluation of the effects of weight-loss intervention.

## CONCLUSION

We used for the first time an open-bore MRI scanner to measure EAT volume in patients with severe/extreme obesity, and we found that a significant EAT loss, both in diastolic and systolic phase, at 12 months after BMS, is associated with BMI and VAT reduction. The absolute change in EAT volume was significantly reduced among wMS patients compared with the MS group. The lack of correlation with MS could be explained by the fact that we measure EAT quantity and not its functional inflammatory status. Moreover, we observed a favorable remodeling of RV and LV after BMS. Consequently, it is likely that the beneficial effects of BMS on EAT are at least partially responsible for the reduced cardiac mortality seen with weight loss. However, future studies are warranted to determine whether the volume or inflammation of EAT is the better cardiac risk measure.

## Figures and Tables

**Fig. (1):**
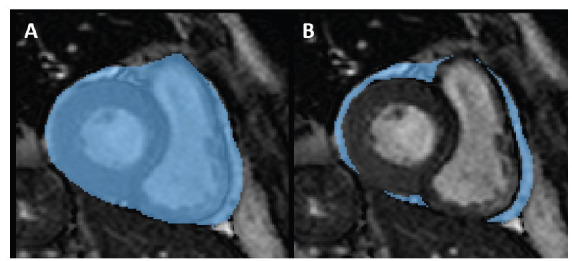
Segmentation of the pericardium (**A**) with subsequent subtraction of the epicardium (**B**) to obtain an estimate of epicardial adipose tissue volume in an image obtained from a cine sequence in short axis acquired on an open-bore MRI scanner in a 44-year-old male patient referred for bariatric surgery.

**Fig. (2):**
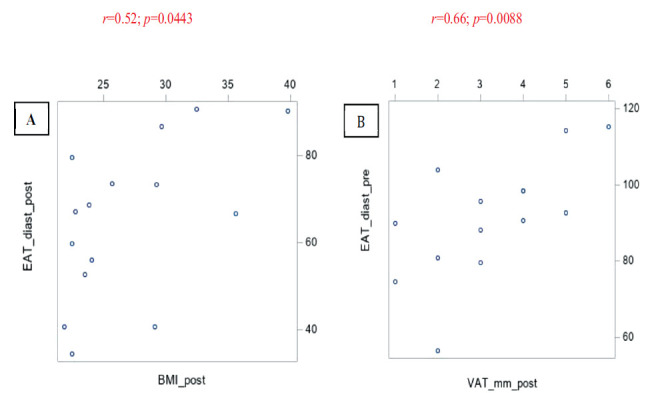
Regression analysis scatter plot shows positive correlation between EAT in the diastolic phase (EAT_diast) and BMI post-BS. (**A**) and between EAT in the diastolic phase (EAT_diast) and VAT (**B**) post-BS.

**Table 1:** Demographic, anthropometric and biochemical characteristic of the study population at baseline. Data were reported as median and interquartile range (IQR).

**Variable**	**Total (N=15)**
Age (years)	47.0 (40.0-55.0)
Weight (Kg)	107.0 (101.00-113)
Height (cm)	161.0 (156.0-164.5)
BMI (kg/m^2^)	41.2 (40.1-42.8)
Waist circumference (cm) F (N=13) M (N=2) TOT (N=15)	117.5 (110.0-121.0) 125.3 (118.0-132.5) 118.0 (110.0-122.0)
WHtR (cm)	0.74 (0.68-0.77)
LAP (cm x mmol/L)	73.95 (46.63-122.67)
Fasting blood glucose (mg/dl)	101.0 (91.0-114.0)
HbA1c (mmol/ml)	37 mmol/mol (34-41)
Total Cholesterol (mg/dl)	194.0 (174.0-228.0)
Triglycerides (mg/dl)	127.0 (71.0-205.0)
HDL-C (mg/dl)	49.0 (44.0-64.0)

**Abbreviations:** BMI: Body Mass Index; WHtR: Waist-to-Height Ratio; LAP: Lipid Accumulation Product; HDL-C: High-density lipoprotein-Cholesterol.

**Table 2:** Changes of the anthropometric and biochemical characteristic after BS: T0 *vs* T1. Data were reported as median and interquartile range (IQR).

**Variable**	**Time**	**Total (N=15)**	**SG (N=9) RYGBP (N=6)**	***p*-value (T0-T1 Total)**	***p*-value (SG *vs* RYGBP T0)**
Weight (Kg)	T0 T1	107.0 (101.0-113.0) 73.0 (62.0-77.0)	103.0(100.0-107.0) 110.5 (107.0-114.0) 74.0 (70.0-75.0) 65.5 (62.0-77.0)	<0.0001	0.1110
BMI (kg/m^2^)	T0 T1	41.2 (40.1-42.8) 24.1 (22.5-29.6)	42.3 (40.4-42.9) 40.8 (39.0-41.9) 29.1 (23.9-29.6) 23.0 (22.5-24.1)	<0.0001	0.1407
Waist circumference (cm)	T0 T1	118.0 (110.0 -122.0) 84.3 (75.4-92.4)	119.0 (117.0-122.0) 117.5 (110.0-121.0) 79.0 (75.4-85.9) 89.9 (77.0-93.4)	<0.0001	0.8135
WHtR (cm)	T0 T1	0.74 (0.68-0.77) 0.54 (0.48-0.56)	0.75 (0.72-0.77) 0.70 (0.67-0.74) 0.49 (0.48-0.54) 0.55 (0.48-0.57)	<0.0001	0.1562
LAP (cm x mmol/L)	T0 T1	73.95 (46.63-122.67) 22.79 (15.44-27.18)	62.30 (46.63-101.81) 104.03(51.96-122.67) 21.10 (15.44-24.26) 24.25 (17.53-27.18)	<0.0001	0.5169
Fasting blood glucose (mg/dl)	T0 T1	101.0 (91.0-114.0) 88.0 (83.0-93.0)	101.0 (87.0-114.0) 103.5 (94.0-113.0) 84.0 (79.8-92.0) 88.0 (88.0-93.0)	0.0037	0.9061
Total Cholesterol (mg/dl)	T0 T1	194.0 (174.0-228.0) 199.0 (177.0-215.0)	194.0 (176.0-201.0) 210.0 (163.0-231.0) 213.0 (182.5-226.0) 179.0 (177.0-199.0)	0.6223	0.8133
Triglycerides (mg/dl)	T0 T1	127.0 (71.0-205.0) 76.0 (64.0-112.0)	89.0 (70.0-131.0) 159.0 (78.0-205.0) (69.5-119.5) 68.0 (63.0-84.0)	0.0139	0.3160
HDL-C (mg/dl)	T0 T1	49.0 (44.0-64.0) 65.0 (55.0-71.5)	50.0 (44.0-68.0) 48.5 (46.0-52.0) 65.0 (61.0-68.0) 70.0 (49.0-78.0)	0.0073	0.5956

**Abbreviations:** BMI: Body Mass Index; WHtR: Waist-to-Height Ratio; LAP: Lipid Accumulation Product; HDL-C: High-density lipoprotein-Cholesterol.

**Table 3:** Changes in EAT and VAT volume after BS: T0 *vs* T1. Data were reported as median and interquartile range (IQR).

**Variable**	**Time**	**Total (N=15)**	**SG (N=9) RYGBP (N=6)**	***P*-value (T0-T1 Total)**	***P*-value (SG *vs* RYGBP T0)**
EAT Diast (cm^3^)	T0 T1	91.6 (80.9-98.3) 67.1 (52.7-79.5)	91.3 (77.1-104.9) 94.4 (88.1-98.3) 68.6 (66.6-73.5) 57.9 (52.7-79.5)	0.0002	0.6588
EAT Syst (cm^3^)	T0 T1	89.4 (78.0-105.7) 68.2 (49.8-75.1)	84.7 (75.1-98.8) 102.6 (81.2-108.5) 68.8 (64.7-75.1) 63.3 (49.8-73.1)	0.0002	0.4166
VAT (mm)	T0 T1	6.45 (4.0-9.0) 3.2 (2.0-4.25)	7.0 (5.0-9.0; 4.0) 4.5 (4.0-5.75) 3.5 (2.25-4.0) 3.0 (2.0-5.0; 3.0)	0.0010	0.3280

**Abbreviations:** EAT: Epicardial Adipose Tissue; VAT: Visceral Adipose Tissue.

**Table 4:** Changes in morpho-funcional parameters after BS: T0 *vs* T1. Data were reported as median and interquartile range (IQR).

**Variable**	**Time**	**Total (N=15)**	**SG (N=9) RYGBP (N=6)**	***p*-value (T0-T1 Total)**
LV EDVi (ml/ m^2^)	T0 T1	54.0 (41.0-57.0) 60.2 (54.5-68.5)	50.0 (41.0-56.0) 54.1 (46.3-64.0) 59.4 (54.5-61.7) 68.0 (59.1-69.8)	0.0009
RV EDVi (ml/m^2^)	T0 T1	49.4 (46.0-58.0) 67.5 (57.6 -74.0)	49.0 (46.0-51.0) 57.3 (49.4-68.0) 62.2 (57.6-67.5) 74.4 (68.0-85.6)	0.0015
LV EF (%)	T0 T1	71.0 (63.0-72.0) 71.6 (69.1-75.6)	71.0 (63.0-72.0) 70.4 (69.3-71.0) 72.4 (69.7-75.6) 70.9 (69.7-71.7)	0.6788
RV EF (%)	T0 T1	66.0 (57.0-71.0) 63.3 (59.5-69.0)	70.0 (64.0-71.0) 60.3 (57.0-70.2) 66.9 (63.2-70.4) 59.9 (58.2-63.3)	0.7615
LV ESVi (ml/m^2^)	T0 T1	16.0 (14.2-18.0) 17.8 (14.7-20.8)	16.0 (15.0-17.0) 15.7 (14.2-18.0) 16.3 (14.7-18.7) 20.0 (17.6-21.6)	0.2078
RV ESVi (ml/m^2^)	T0 T1	17.0 (14.0-23.8) 24.8 (18.0-30.1)	14.0 (14.0-21.0) 21.4 (14.7-24.0) 19.7 (17.9-24.8) 29.3 (26.7-35.8)	0.0006
LV mass index (g/ m^2^)	T0 T1	59.0 (50.0-63.0) 61.8 (54.7-70.6)	52.0 (50.0-62.0) 60.2 (59.0-73.0) 61.2 (54.6-61.8) 72.3 (62.9-76.2)	0.2238
RV mass index (g/m^2^)	T0 T1	29.0 (24.0-31.0) 38.2 (32.8-44.4)	29.0 (24.0-31.0) 28.8 (27.0-30.4) 37.2 (32.7-42.3) 43.2 (35.0-44.4)	0.0001
LV SV (ml)	T0 T1	75.0 (60.0-83.0) 78.1 (70.0-82.7)	72.0 (60.0-79.0) 81.3 (65.2-84.0) 74.9 (68.0-80.0) 82.2 (72.9-87.9)	0.4212
RV SV (ml)	T0 T1	70.0 (59.0-75.0) 75.4 (68.8-83.5)	67.0 (59.0-72.0) 70.8 (70.0-85.0) 72.4 (65.6-76.1) 82.8 (75.4-83.5)	0.0833
Area min (mm)	T0 T1	511.0 (433.0-634.0) 554.1 (487.3-611.3)	549.2 (491.0-634.0) 484.6 (424.2-511.0) 554.1 (486.9-603.5) 566.4 (487.3-632.8)	0.6848
Area max (mm)	T0 T1	603.3 (551.0-753.0) 652.5 (587.3-742.4)	643.0 (603.3-753.0) 576.7 (551.0-526.3) 652.5 (587.3-726.0) 691.3 (560.8-808.0)	0.6848
AAS	T0 T1	0.21 (0.18-0.28) 0.20 (0.16-0.21)	0.21 (0.19-0.28) 0.21 (0.16-0.23) 0.18 (0.15-0.20) 0.22 (0.18-0.28)	0.6355

**Abbreviations:** LV EDVi: left ventricular end-diastolic volume index; RV EDVi: right ventricular end-diastolic volume index; LV ESVi: left ventricular end-sistolic volume index; RV ESVi: right ventricular end-sistolic volume index; LV VF: left ventricular ejection fraction; RV EF: right ventricular ejection fraction; LV SV: left ventricular stroke volume; RV SV: right ventricular stroke volume; AAS: Aortic Area Strain.

**Table 5:** Comparison between changes in EAT volume and VAT after BS in patients with and without Metabolic Syndrome (MS *vs* wMS). Data were reported as median and interquartile range (IQR).

**Variable**	**Time**	**Total (N=15)**	**MS (N=7) wMS (N=8)**	***p*-value**		
**-**	**-**	**-**	**-**	**MS and wMS**	**T0-T1 MS**	**T0-T1 wMS**
EAT Diast (cm^3^)	T0 T1	91.6 (80.9-98.3) 67.1 (52.7-79.5)	98.3 (92.6-114.3) 80.9 (74.6-90.6) 79.5 (67.1-90.0) 54.4 (40.7-64.1)	0.0152	0.031 0.00065	0.0156
EAT Syst (cm^3^)	T0 T1	89.4 (78.0-105.7) 68.2 (49.8-75.1)	105.7 (82.4-112.9) 81.2 (74.4-99.8) 75.1 (69.9-86.3) 54.1 (40.8-65.9)	0.0967	0.031 0.0015	0.0156
VAT (mm)	T0 T1	6.45 (4.0-9.0) 3.2 (2.0-4.25)	7.0 (5.0-10) 5.0 (4.0-7.5) 4.0 (2.5-5.0) 3.0 (2.0-4.0)	0.27	0.036 0.41	0.021

**Abbreviations:** EAT: Epicardial Adipose Tissue; VAT: Visceral Adipose Tissue.

**Table 6:** Comparison between changes in anthropometric and biochemical variable after BS in patients with and without Metabolic Syndrome (MS *vs* wMS). Data were reported as median and interquartile range (IQR).

**Variable**	**Time**	**MS (N=7) wMS (N=8)**	***p*-value**		
**-**	**-**	**-**	**MS *vs* wMS**	**T0-T1 MS**	**T0-T1 wMS**
Weight (Kg)	T0 T1	110.0 (101.0-114.0) 105.0 (100.0-107.5) 77.0 (74.0-89.0) 65.5 (61.5-71.5)	0.2968	0.0156 0.0205	0.0078
BMI (kg/m^2^)	T0 T1	40.9 (39.7-43.4) 41.6 (40.3-42.6) 29.6 (22.8-35.7) 23.7 (22.5-26.6)	0.8622	0.0156 0.1049	0.0078
Waist circumference (cm)	T0 T1	121.0 (119.0-129.0) 113.5 (108.5-117.3) 79.0 (73.3-92.4) 85.1 (76.2-89.9)	0.0128	0.0156 0.8168	0.0078
WHtR (cm)	T0 T1	0.74 (0.72-0.80) 0.71 (0.68-0.76) 0.49 (0.43-0.59) 0.54 (0.49-0.55)	0.3532	0.0156 0.7270	0.0078
LAP (cm x mmol/L)	T0 T1	122.34 (62.30-154.96) 49.30 (44.96-79.84) 21.10 (14.06-31.10) 23.41 (16.49-24.37)	0.0562	0.0156 0.6854	0.0078
Fasting blood glucose (mg/dl)	T0 T1	111.0 (101.0-121.0) 92.5 (85.0-105.0) 92.0 (80.0-97.0) 86.0 (83.0-88.0)	0.0560	0.0625 0.3254	0.0469
Total Cholesterol (mg/dl)	T0 T1	194.0 (174.0-201.0) 210.0 (169.5-237.0) 213.0 (191.0-218.0) 179.0 (174.0-215.0)	0.5621	0.0625 0.5197	0.0781
Triglycerides (mg/dl)	T0 T1	172.0 (89.0-255.0) 78.0 (68.5-138.5) 80.0 (75.0-123.0) 68.0 (61.0-112.0)	0.0930	0.1563 0.2246	0.1094
HDL-C (mg/dl)	T0 T1	44.0 (30.0-50.0) 55.0 (47.5-66.0) 61.0 (49.0-68.0) 67.5(65.0-78.0)	0.0823	0.031 0.2963	0.1563

**Abbreviations:** BMI: Body Mass Index; WHtR: Waist-to-Height Ratio; LAP: Lipid Accumulation Product; HDL-C: High-density lipoprotein-Cholesterol.

**Table 7:** Comparison between changes in cardiac morpho-funcional parameters after BS in patients with and without Metabolic Syndrome (MS *vs* wMS). Data were reported as median and interquartile range (IQR).

**Variable**	**Time**	**Total (N=15)**	**MS (N=7) wMS (N=8)**	***p*-value**		
**-**	**-**	**-**	**-**	**MS and wMS**	**T0-T1 MS**	**T0-T1 wMS**
LV EDVi (ml/m^2^)	T0 T1	54.0 (41.0-57.0) 60.2 (54.5-68.5)	41.0 (35.0-56.0) 54.1 (52.0-59.0) 60.2 (51.2-64.6) 63.5 (59.0-69.2)	0.0930	0.0156 0.4519	0.0547
RV EDVi (ml/m^2^)	T0 T1	49.4 (46.0-58.0) 67.5 (57.6 -74.0)	47.0 (37.0-58.0) 54.0 (49.2-62.8) 62.2 (58.4-68.7) 70.3 (62.5-80.0)	0.1645	0.0156 0.3253	0.0781
LV EF (%)	T0 T1	71.0 (63.0-72.0) 71.6 (69.1-75.6)	71.0 (56.0-78.3) 71.0 (69.6-71.0) 72.4 (70.9-75.6) 69.6 (65.3-78.0)	0.9530	0.5781 0.2716	0.9453
RV EF (%)	T0 T1	66.0 (57.0-71.0) 63.3 (59.5-69.0)	62.0 (52.0-70.0) 70.1 (64.2-71.5) 66.9 (63.1-70.4) 61.9 (57.9-66.8)	0.1321	0.1094 0.2716	0.0234
LV ESVi (ml/m^2^)	T0 T1	16.0 (14.2-18.0) 17.8 (14.7-20.8)	16.0 (8.0-18.0) 15.7 (15.0-17.5) 15.9 (13.5-18.7) 19.8 (17.7-21.5)	0.6838	0.5781 0.0933	0.1953
RV ESVi (ml/m^2^)	T0 T1	17.0 (14.0-23.8) 24.8 (18.0-30.1)	14.0 (14.0-27.0) 18.0 (14.4-23.4) 18.2 (17.4-25.2) 27.6 (19.8-31.7)	0.8153	0.0469 0.1182	0.0156
LV SV (ml)	T0 T1	75.0 (60.0-83.0) 78.1 (70.0-82.7)	60.0 (56.0-80.0) 79.3 (71.0-86.0) 80.0 (73.9-82.7) 73.9 (69.5-84.8)	0.1179	0.0781 0.5244	0.2500
LV mass index (g/m^2^)	T0 T1	59.0 (50.0-63.0) 61.8 (54.7-70.6)	59.0 (50.0-63.0) 58.5 (51.0-68.2) 61.2 (52.9-61.8) 63.0 (61.8-72.3)	0.8166	0.6875 0.1182	0.3828
RV SV (ml)	T0 T1	70.0 (59.0-75.0) 75.4 (66.8-83.5)	59.0 (51.0-75.0) 70.8 (68.5-74.5) 76.1 (72.4-88.2) 73.7 (66.2-83.4)	0.1480	0.0313 0.5244	0.9453
RV mass index (g/m^2^)	T0 T1	29.0 (24.0-31.0) 38.2 (32.7-44.4)	31.0 (25.7-33.0) 27.8 (23.5-29.5) 38.2 (36.4-47.2) 38.5 (28.2-43.3)	0.1318	0.0156 0.2712	0.0156
Area min (mm)	T0 T1	511.0 (433.0-634.0) 554.1 (487.3-611.3)	560.0 (511.0-672.0) 453.5 (375.6-517.6) 563.3 (537.1-632.8) 504.2 (470.9-611.3)	0.0177	0.2969 0.2840	0.2188
Area max (mm)	T0 T1	603.3 (551.0-753.0) 652.5 (587.3-742.4)	661.0 (603.3-824.0) 556.7 (490.5-619.6) 653.7 (615.6-808.0) 613.8 (529.1-742.4)	0.0206	0.6875 0.3914	0.3125
AAS	T0 T1	0.21 (0.18-0.28) 0.20 (0.16-0.21)	0.22 (0.16-0.28) 0.21 (0.19-0.28) 0.18 (0.15-0.20) 0.21 (0.18-0.23)	0.6025	0.4688 0.2840	1.000

**Abbreviations:** LV EDVi: left ventricular end-diastolic volume index; RV EDVi: right ventricular end-diastolic volume index; LV ESVi: left ventricular end-sistolic volume index; RV ESVi: right ventricular end-sistolic volume index; LV VF: left ventricular ejection fraction; RV EF: right ventricular ejection fraction; LV SV: left ventricular stroke volume; RV SV: right ventricular stroke volume; AAS: Aortic Area Strain.

## Data Availability

The datasets generated and analyzed during the current study are available from the corresponding author upon reasonable request.

## LIST OF ABBREVIATIONS

AAS: Aortic Area Strain
BMI: Body Mass Index
BMS: Bariatric and Metabolic Surgery
CAD: Coronary Artery Disease
CT: Computed Tomography
EAT: Epicardial Adipose Tissue
ECG: Electrocardiographic
EDV: End-diastolic Volume
EF: Ejection Fraction
ESV: End-systolic Volume
FBG: Fasting Blood Glucose
HDL-C: High-density Lipoprotein Cholesterol
INCO: National Institute for Obesity Treatment
LAP: Lipid Accumulation Product
LSG: Laparoscopic Sleeve Gastrectomy
LV EDVi: Left Ventricular End-diastolic Volume Index
MRI: Magnetic Resonance Imaging
MS: Metabolic Syndrome
NCEP/ATPIII: National Cholesterol Education Program/Adult Treatment Panel III Criteria
RV EDVi: Right Ventricular End-diastolic Volume Index
RYGBP: Laparoscopic Gastric By-pass According to Roux-en-Y
SGLT2i: Sodium-glucose Co-transporter Inhibitors
SV: Stroke Volume
TC: Total Cholesterol
TGs: Triglycerides
VAT: Visceral Adipose Tissue
WC: Waist Circumference
WHtR: Waist-to-height Ratio

## STANDARDS OF REPORTING

SAGER guidelines were followed.
